# Genomic analyses of early peri-implant bone healing in humans: a systematic review

**DOI:** 10.1186/s40729-015-0006-2

**Published:** 2015-03-01

**Authors:** Siddharth Shanbhag, Vivek Shanbhag, Andreas Stavropoulos

**Affiliations:** 1 Department of Periodontology, Faculty of Odontology, Malmö University, Carl Gustafs väg 34, 214 21 Malmö, Sweden; 2 Centre for Oral Rehabilitation & Implant Dentistry, 1 Laxmi Niwas, 87 Bajaj Road, Vile Parle West, Mumbai, 400056 India

**Keywords:** Dental implant, Osseointegration, Gene expression, Molecular assessment

## Abstract

**Objective:**

The objective of the study was to systematically review the literature for studies reporting gene expression analyses (GEA) of the biological processes involved in early human peri-implant bone healing.

**Methods:**

Electronic databases (MEDLINE, EMBASE) were searched in duplicate. Controlled and uncontrolled studies reporting GEA of human peri-implant tissues - including ≥5 patients and ≥2 time points - during the first 4 weeks of healing were eligible for inclusion. Methodological quality and risk of bias were also assessed.

**Results:**

Four exploratory studies were included in reporting GEA of either tissues attached to SLA or SLActive implants after 4 to 14 days or cells attached to TiOBlast or Osseospeed implants after 3 to 7 days. A total of 111 implants from 43 patients were analyzed using validated array methods; however, considerable heterogeneity and risk of bias were detected. A consistent overall pattern of gene expression was observed; genes representing an immuno-inflammatory response were overexpressed at days 3 to 4, followed by genes representing osteogenic processes at day 7. Genes representing bone remodeling, angiogenesis, and neurogenesis were expressed concomitantly with osteogenesis. Several regulators of these processes, such as cytokines, growth factors, transcription factors, and signaling pathways, were identified. Implant surface properties seemed to influence the healing processes at various stages via differential gene expression.

**Conclusion:**

Limited evidence from gene expression studies in humans indicates that osteogenic processes commence within the first post-operative week and they appear influenced at various stages by implant surface properties.

## Review

### Introduction

Osseointegrated oral implants are an integral part of modern reconstructive dentistry and are associated with favorable long-term therapeutic outcomes [1]. Osseointegration was originally defined as the direct contact between vital bone and a load-bearing implant observed at the light microscopic, i.e., histological, level [2]. Morphogenesis of implant osseointegration has been assessed in several preclinical *in vivo* and clinical histological studies [3-6], providing the basis for understanding the biological process.

The biological events during the early phase of osseointegration are directly influenced by the osseous microenvironment (i.e., cells, signaling molecules, and matrix) into which the implant is placed and have many similarities with general wound healing mechanisms [7]. Implant surgery induces trauma, resulting in bleeding and fibrin clot formation and an inflammatory reaction that dominate the events of the first post-operative week. The deposition of vital new bone on the implant surface by osteoblasts (osteogenesis), a fundamental requirement for osseointegration, occurs via secretion of a complex extracellular matrix (ECM) of proteins, which subsequently undergoes mineralization to form bone [8,9]. Primary (woven) bone lined by osteoblasts can indeed be observed on the implant surface already after 1 week [3,5]. In parallel, removal of the created bone debris and remodeling of necrotized bone (due to the pressure exerted by the implant) is underway. Replacement of woven bone by organized and mechanically superior lamellar bone can be observed from the second to fourth week (depending on the species) and progressively increases until woven bone is almost entirely replaced (8 to 12 weeks). These events, including the nutrition of the newly formed tissue, are sustained through concomitantly occurring angiogenesis, i.e., formation of new blood vessels from existing ones [10,11]. Thus, osseointegration is a dynamic process whereby bone formation and remodeling occur in parallel around the implant [4,6].

Morphogenesis of osseointegration and assessment of the degree of bone-to-implant contact is usually performed by means of histological evaluation [12], while the underlying molecular processes may be more precisely evaluated at genetic level [13,14]. Data from gene expression analyses of fracture healing provide the basis for understanding these processes [15]. These studies have identified the cells, signals, and interactions governing the key processes of bone regeneration. Bone-forming osteoblasts are primarily derived from marrow-resident multipotent progenitor cells (mesenchymal stem cells (MSCs)), which are recruited to the regeneration site. This process of MSC recruitment and differentiation along the osteogenic lineage is termed as osteoinduction and is controlled primarily by various pro/anti-inflammatory cytokines (CKs) and by growth factors (GFs) secreted by inflammatory cells and/or osteoblasts or by GF resident within the extracellular matrix (e.g., bone morphogenetic proteins (BMPs)) in response to injury [16-18]. Moreover, CKs and GFs act as signaling molecules via specific signaling pathways and guide the process of cell differentiation in the proper temporal sequence [19,20]. Intermediaries in this process are various bone-specific transcription factors (TFs), which act as ‘molecular switches’ during cell differentiation and are targets of CKs and GFs [21]. TFs facilitate bone-specific gene transcription and ultimately gene expression by which MSCs undergo differentiation and acquire the osteoblastic phenotype [22]. While GFs regulate mainly osteoinduction and osteogenesis, pro-inflammatory CKs regulate the antagonist process of bone resorption by inducing the differentiation of hematopoietic stem cells (HSCs) into osteoclasts and macrophages [23], contributing to the dynamic nature of bone regeneration and remodeling.

Recent *in vitro* [24] and preclinical *in vivo* [25] studies have focused on the early molecular biological responses to various titanium implant surfaces. Understanding these early responses is essential for efforts aiming to accelerate and enhance the process of osseointegration [26]. Upregulation or downregulation of specific genes in peri-implant tissues identified by analyses of genetic material (DNA, RNA) reflects the nature and timing of the various healing processes, which in turn could be potential ‘molecular targets’ for enhancing osseointegration [27,28]. The aim of the present study was to systematically review the available literature on gene expression analyses of the biological processes involved in early human peri-implant bone healing.

## Methods

### Study design

A study protocol for a systematic qualitative literature review was developed based on recommended methods [29]. The focused question was ‘what biological processes are reflected by gene expression analyses in peri-implant tissues of humans during the early stages (up to 4 weeks) of healing?’

### Inclusion and exclusion criteria

All studies, controlled (using different implants) or uncontrolled, reporting gene expression analyses of peri-implant tissues harvested from ≥5 human patients at ≥2 time points during the first 4 weeks of healing, were eligible for inclusion. Studies reporting the use of either ‘experimental’ (micro) or standard implants with clear description of implant surface properties, placed in the maxilla or mandible and retrieved at a later time point, were eligible for inclusion. Studies reporting (1) analyses of peri-implant mucosa or sulcular fluid or peri-implant tissues of failing or infected implants (peri-implantitis), (2) only histological or immunohistochemical analyses without gene expression of harvested tissues, and (3) *in vitro* and preclinical *in vivo* studies were excluded. Primary outcome of interest was the biological process (or processes) reflected by gene expression at a particular time point of peri-implant tissue healing.

### Search strategy

Electronic databases of MEDLINE (via PubMed) and EMBASE were searched by one author (SS) for relevant English-language literature up to and including June 2014. The search strategy used for MEDLINE was ((((("gene expression" OR transcriptome OR transcriptional OR molecular OR microarray))) AND ((osseointegration OR healing OR "peri implant"))) AND implants) AND ((human OR humans OR patients OR subjects)). Unpublished literature was searched via the Google and Google Scholar search engines. Additionally, the bibliographies of all relevant studies and review articles were searched.

### Study selection

Titles and abstracts of the search identified studies were screened by two authors (SS and VS) based on the inclusion criteria, and full texts of all eligible studies were obtained. Differences in assessment of eligibility were resolved by discussion with the third author (AS). Full texts were independently reviewed by both reviewers, and final inclusion was based on the aforementioned inclusion criteria.

### Data extraction

Both reviewers independently extracted data from the full texts of included articles using specially designed forms. Data on author(s), study design, implant type/surface, any additional procedures performed, number of patients (in each group), presence of a control group, procedure and time of implant retrieval, methods of gene expression analysis, and main results, were extracted. Descriptive summaries of the studies were entered into tables, and a qualitative synthesis of evidence was planned. Any disagreement between the reviewers regarding data extraction was resolved by discussion.

### Assessment of methodology and risk of bias

Assessment of the methodological validity of the included studies was performed using criteria adapted from previous reports [30,31]. Aspects of study design, genotyping methods, and data analyses were considered using nine criteria (Table 1).

**Table 1:** **Assessment of the genotyping methodology in the included studies**

**Methodology**	**Ivanovski et al. [** 34 **]**	**Donos et al. [** 35 **]**	**Bryington et al. [** 36 **]**	**Thalji et al. [** 37 **]**
Tissue harvesting	Tissue attached to implant carefully removed with a curette, preexisting hard tissue discarded	Tissue attached to implant carefully removed with a curette and homogenized	Implants removed by reverse threading and homogenized; cell lysates isolated	Implants removed by reverse threading and homogenized; cell lysates isolated
Sample preparation	Total RNA isolation, purification, quantity/quality analysis and biotin-labeling	Total RNA isolation, purification, quantity/quality analysis and biotin-labeling	Total RNA isolation, quantification	Total RNA isolation, purification, quantity/quality analysis and biotin-labeling
Array technique	Microarray hybridization (Human WG-6 V3)	Microarray hybridization (Human WG-6 V3)	RT-PCR (custom RT-PCR array for osteogenesis genes; human inflammatory cytokines and receptors PCR array)	Microarray hybridization (Affymetrix Human Gene 1.1 ST)
Scanning, data preparation	Bead Station 500/Bead Studio v3 software, raw probe expression values extracted	Bead Station 500/ Bead Studio v3 software, raw probe expression values extracted	RT^2^ SYBR Green qPCR Master Mix/7500 Real-Time PCR system	Affymetrix Gene Chip Scanner
Processing	Noisy data discarded	Noisy data discarded	Normalization of osteogenesis and cytokine array	Unclear
Clustering	GO categories (DAVID tool)	GO categories (DAVID tool)	Osteogenesis genes; cytokine-related genes	GO categories (Gene Spring)
Statistical analysis	Gene Spring software	Gene Spring software	RT^2^ Profiler software	Gene Spring software
Comparisons	Pair-wise comparisons between three time points (4 vs. 7 days, 7 vs. 14 days, and 4 vs. 14 days)	Pair-wise comparisons at each time point (4, 7, and 14 days) between SLA and SLActive surfaces	T-test to evaluate differences between each implant surface per time point	Two-way ANOVAs to determine differences between implant surface type and time points; pair-wise comparisons of each implant surface independently at different time points (day 7 vs. day 3)

GO, gene ontology; DAVID, Database for Annotation, Visualization and Integrated Discovery.

The risk of bias in the included studies was assessed using an adaptation of published guidelines for reporting systematic reviews of periodontal genetic association studies [32]. Mainly, aspects of study design and methodological validity were assessed using 15 criteria and scored as ‘yes,’ ‘no,’ or ‘unclear’ based on the information provided in the study manuscript (Table 2). Moreover, published guidance [33] regarding the qualitative and quantitative syntheses of results from genetic association studies was consulted, and heterogeneity across the included studies was assessed to explore the possibility of a meta-analysis.

**Table 2:** **Assessment of risk of bias and heterogeneity within and across the included studies**

**Category**	**Ivanovski et al. [** 34 **]**	**Donos et al. 2011 [** 35 **]**	**Bryington et al. [** 36 **]**	**Thalji et al. [** 37 **]**
Study design				
Comparison	None (only SLActive)	SLA vs. SLActive	TiOBlast vs. Osseospeed	TiOBlast vs. Osseospeed
Setting	University	University	University	University
Population, inclusion criteria	9 healthy volunteers with no mandibular third molars, no contraindications for oral surgery; age 21 to 48, median 29 years	9 healthy volunteers with no mandibular third molars; age 21 to 48, median 29 years	6 women, 4 men; implant patients, systemically healthy (no HTN, diabetes, CVD); age 25 to 58, mean 36.2 years	9 women, 2 men; implant patients, systemically healthy; age 47 to 69, mean 60.2 years
Exclusion criteria	Smokers	Smokers	Smokers, pregnancy, periodontal/periapical disease, subjects taking bisphosphonates, hormone replacement therapy, corticosteroids	Smokers, uncontrolled diabetes, history of head/neck radiotherapy, taking corticosteroids, bisphosphonates
Comparability of groups	Unclear	Unclear	Unclear	Unclear
Potential confounders, e.g., post-op medication	Unclear	Unclear	Unclear	Unclear
Power calculation	No	No	No	No
Statistical correction	For multiple sampling	For multiple sampling	Unclear	For multiple sampling
Methods				
Tissue analyzed	Peri-implant tissue	Peri-implant tissue	Implant-adherent cells	Implant-adherent cells
Genetic material analyzed	Total RNA	Total RNA	Total RNA	Total RNA
Success rate	Unclear	16/18 samples (88.8%)	7/10 subject samples (70%)	Unclear
Genotyping method	Microarray	Microarray	RT-PCR	Whole-genome microarray
Genotype counts	Yes	Yes	Yes	Yes
Blinding	Unclear	Unclear	Yes	Unclear
Reproducibility, validated genotyping accuracy	No	No	No	No

All studies were judged to be at a high risk of bias with substantial heterogeneity across studies.

## Results and discussion

The included studies basically report on commercially available implants from two major manufacturers and involve comparisons of different implant surface technologies in regard with topography and/or chemistry modifications within each implant system. Various analyses were performed in the included studies; however, an attempt has been made to synthesize the various findings and discuss them herein irrespective of the specific implant systems, based on the assumption that basic biological mechanisms of peri-implant bone wound healing are largely implant system independent.

### Search results and study characteristics

**Figure 1:**
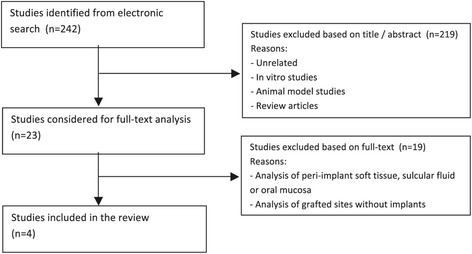
**Flowchart for study selection (** ***n*** **= number of studies).**

Of the 242 search identified studies, only four studies were finally included in the review, all focusing on the impact of implant surface on early human peri-implant bone healing (Figure 1; Table 3). Genetic analyses of total RNA isolated from either newly formed peri-implant bone harvested by trephination [34,35] or from cells adherent to implants retrieved by reverse threading [36,37] were performed. In total, 111 implants from 43 patients were analyzed. All four studies reported the use of commercially existing implant surfaces, i.e., either a chemically modified, hydrophilic, sand-blasted, acid-etched surface (SLActive®, Institute Straumann AG, Basel, Switzerland); or a hydrophilic (SLActive®) versus a hydrophobic unmodified SLA® (Institute Straumann AG, Basel, Switzerland) surface; or a micro-topographic titanium-oxide grit-blasted surface (TiOBlast®, AstraTech, Molndal, Sweden) versus a chemically modified nano-topographic grit-blasted surface (Osseospeed®, AstraTech, Molndal, Sweden). Implant retrieval times were at 3 or 4 days and 7 days in all studies and additionally at 14 days in two studies [34,35].

**Table 3:** **Summary of findings from the included studies (** ***n*** **= 4)**

**Study**	**Ivanovski et al. [** 34 **]**	**Donos et al. [** 35 **]**	**Bryington et al. [** 36 **]**	**Thalji et al. [** 37 **]**
Design	9 patients; 9 implants placed	18 patients; 18 implants placed	10 patients; 60 implants placed	11 patients; 44 implants placed
	Total RNA extracted from peri-implant tissue (trephine)	16 samples analyzed	42 samples analyzed	Total RNA extracted from implant adherent cells (reverse thread)
		Total RNA extracted from peri-implant tissue (trephine)	Total RNA extracted from implant adherent cells (reverse thread)	
Surface	SLActive	SLA vs. SLActive	TiOBlast vs. Osseospeed	TiOBlast vs. Osseospeed
GE day 3/4	Upregulated	Upregulated on SLA	Upregulated on both surfaces	No significant differences between surfaces at any time point (*P* > 0.05)
	CKs (TNF-a, IL-6, IL-2)	Neurogenesis	Osteogenesis (Runx2, Osx, BMP6, OPN)	Results presented as GE at day 7 vs. day 3 for each surface
	Immune-inflammatory cells (LC, MP)	Collagen organization	Inflammatory CKs (IL-1A,B, TNF)	
	Inflammatory NF-kB p/w	Upregulated on SLActive	MP activity	
		Ras protein p/w	Upregulated on Osseospeed	
		Collagen organization	Chemotaxis (CCL18, CXCL10, CXCL14)	
		CK response	Anti-inflammatory CKs (TOLLIP, IL9, IL22)	
GE day **7**	Upregulated	Upregulated on both surfaces	Upregulated on both surfaces	Upregulated on both surfaces
	MSC genes (HOX, Sp3)	Inflammatory CKs (IL1, IL2, IL6, TNFS)	Osteogenesis (Runx2, Osx*, OCN*, OPN, BMP6, BSP)	ECM (Coll, GPs, PGs)
	GF (TGF-B receptor)	Neurogenesis	[* Osseo > TiOB; *P* < 0.05]	Collagen organization (PLODs, LOX, PCOLCE)
	VEGF sig. (vs. day 14)	Upregulated on SLActive		Angiogenesis/VEGF sig. (ANXA, EPAS1)
	Wnt p/w	Neurogenesis (BDNF, NTF3)		Ossification
	Downregulated	ECM (OPN)		Remodeling (MMPs, TIMPs)
	Inflammatory NF-kB p/w (vs. day 4)	BMP p/w (BMP4, BMP2K)		Osteoclastic (CTSK, ACP5)
		MAPK sig.		Chemotaxis (CKs, MP activity)
		Mineralization		Anti-inflammatory CKs (CCL22, CCL18)
		Focal adhesion (integrins)		Downregulated on both surfaces
		Angiogenesis (VEGF sig., P13-Akt p/w)		Inflammatory CKs (IL1A, IL1B)
		Downregulated on SLActive		
		Inflammatory cells (LC)		
GE day 14	Upregulated	Upregulated on both surfaces	-	
	ECM (Coll, OC, ON, ALP)	BMP p/w (BMP4, BMP2K)		
	TFs (Osx, Dlx5, Twist1, Smad6)	Downregulated on both surfaces		
	Remodeling (MMP, CTSK)	Inflammatory cells (LC)		
	GFs (BMP, GDF)			
	Angiogenesis (VEGF sig.)			
	Neurogenesis			
	TGF-b/BMP, Notch p/w			
	Ras protein p/w			
	Wnt-receptor genes			
	Notch genes (up/down)			
	Downregulated			
	Inflammatory response (vs. day 7)			

GE, gene expression; CKs, cytokines; p/w, pathway; MSC, mesenchymal stem cells; GF, growth factors; sig., signaling; ECM, extracellular matrix; TFs, transcription factors; MP, macrophage; LC, lymphocytes; GPs, glycoproteins; PGs, proteoglycans.

### Assessment of methodology and risk of bias

All studies used validated methods for gene expression analysis; genetic data was analyzed using microarray (three studies) or real-time PCR (RT-PCR) (one study) methods (Table 1). Total RNA was isolated from lysates of either trephined peri-implant tissues or implant-adherent cells, and subjected to microarray processing or RT-PCR. Although moderate-to-good agreement has been reported between the two methods, validation of DNA microarray results by the more sensitive PCR array is generally recommended [38]. None of the microarray studies identified have validated their results using RT-PCR. Genotyping data (gene lists) were imported and analyzed using computer software and further condensed into functionally and biologically relevant categories. Nevertheless, differential gene expression in relation to a particular cell type or region of tissue analyzed was not performed [35]. Gene ‘upregulation’ was reported when genes were expressed at a higher level on one implant surface in comparison to another; in context, differentiation between gene expression and over-expression may be difficult to define. Statistical methods were used to compare differences in gene expression between different time points and/or implant surfaces (*P* < 0.05 significance level), while correcting for possible errors, i.e., false gene discovery rate due to multiple sampling [39]. There was considerable heterogeneity across the included studies in terms of study design, population, implant surface technology, genotyping methods, and data analyses (Table 2). Therefore, no meta-analysis of association between gene expression and implant surface properties was relevant.

Thus, high risk of bias should be considered when interpreting the results, due to the above methodological limitations and the overall limited information (four studies) available.

### Biological processes identified through gene expression in peri-implant tissues

**Figure 2:**
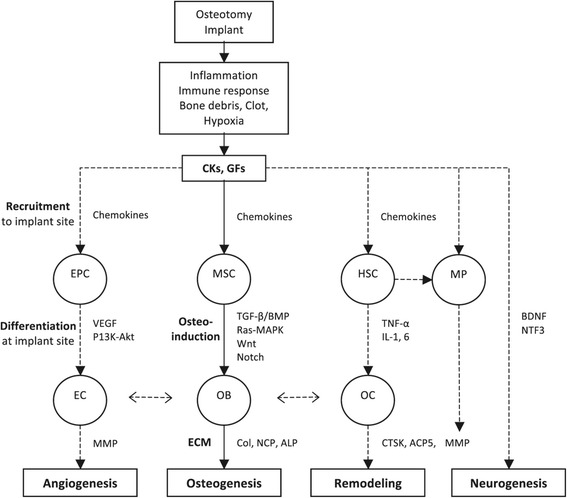
**Summary of biological processes identified via gene expression during early peri-implant bone healing.** CKs, cytokines; GFs, growth factors; EPC, endothelial progenitor cells; EC, endothelial cells; MSC, mesenchymal stem cells; OB, osteoblasts; ECM, extracellular matrix; HSC, haematopoietic stem cells; MP, macrophages; OC, osteoclasts.

Conventional implant surgery involves osteotomy preparation and insertion of the implant into the alveolar bone. The immediate local effects of this procedure, functionally relevant to subsequent healing processes, are (1) bone trauma, (2) formation of bone debris, (3) hemostasis and clot formation, and (4) hypoxia. These effects involve the release of specific CKs and GFs within the local environment [7], resulting in recruitment of two primary cell types to the site, inflammatory cells and progenitor cells (MSCs and HSCs) [19], which in turn regulate the subsequent healing processes. A summary of differentially regulated genes relating to the involved biological processes is presented in Table 4, while Figure 2 represents an evidence-based illustrative model summarizing these processes.

**Table 4:** **Summary of biological processes and associated genes reported in the included studies**

**Process**	**Upregulated genes**
	**Category (gene code)**
Inflammation/immune response	
Pro-inflammatory cytokines	Tumor necrosis factor (TNF-a, TNFSF9)
	Interleukin (IL-6, IL-2, IL-1 F9, IL-23A, IL-6ST)
	Interferon (IFNA2)
	Nuclear factor-kB (I-kB kinase/NF-kB)
Anti-inflammatory cytokines	Interleukin (IL-22, IL-9)
	Toll interacting protein (TOLLIP)
Cells	Lymphocyte, macrophage negative proliferation (BTLA, LST1)
	Macrophage scavenger receptor (MSR1)
Chemotaxis	Chemokines (CCR8, CCL18, CCL22, CXCL10, CXCL14)
Osteoinduction/osteogenesis	
Growth factors (GF)/signaling pathways	Insulin-like GF (IGF1)
	Transforming GF (TGF-b, TGF-b receptor 1, 2 and 3, TGF-a)
	Platelet-derived GF (PDGF receptor)
	Bone morphogenetic proteins (BMP4, BMP6, BMP receptor 1A, BMP2-K)
	Growth and differentiation factor (GDF10)
	Wnt frizzled receptor (FZD3, FZD8, FRZB)
	Notch (NOTCH2)
	Ras-protein signal transduction (RAP1B, RAP1A, RASGRP4)
	Mitogen activated protein kinase (MAP3K7IP2, MAPK9, MAP2K3, MAP3K2)
Transcription factors	‘Master switches’ [RUNX2, SP7 (OSX)]
	Homeobox (DLX1, DLX5, HOXD12, MSX1, HOXA5, HOXB1, HOXB6, HOXC6)
	SP [SP1, SP3, SP7 (Osx)]
	Twist (TWIST 1-receptor)
ECM deposition/organization	Collagen (Col1A1, Col12A1, Col6A3, Col3A1, Col6A1, Col11A1, Col11A2, Col13A1, Col5A2)
	Non-collagen proteins [BGLAP (OC), SPARC (ON), SPP1 (OP), BSP, IBSP, POSTN, ECM1]
	Small leucine-rich proteoglycans (SLRP) (DCN, BGN, LUM)
	Heat-shock protein 47 (HSP47)
	Alkaline phosphatase (ALPL)
	Cadherin (CDH11)
	Integrin (ITGB4, ITGB5)
	Laminin (LAMA2, LAMA3)
	Pro-collagen lysyl-hydroxylase (PLOD1, PLOD2, PLOD3)
	Pro-collagen C-endopeptidase enhancer (PCOLCE)
	Lysyl-oxidase (LOX)
Osteoclast activity/remodeling	Cathepsin K (CTSK, CTSK-receptor)
	Tartarate-resistant acid phosphatase (TRAP/ACP5)
	Matrix metallopeptidase (MMP2, MMP12, MMP9, MMP7, MMP13)
	Tissue inhibitor metallopeptidase (TIMP2, TIMP3)
Angiogenesis	Vascular endothelial GF-signaling (EPAS1, ANXA2, EGR1-binding protein)
	Phosphatidyl-inositol 3-kinase (PI3K)-Akt signaling
Neurogenesis	Brain-derived neurotrophic factor (BDNF)
	Neurotrophin 3 (NTF3)
	NK2 homeobox 2(NKX2-2)
	Tubby-like protein 3 (TULP3)

### Inflammation

All studies reported a significant upregulation of genes associated with inflammation during the first time point of observation (day 3 or 4) regardless of the implant surface. Specifically, upregulation regarded pro-inflammatory cytokines of the interleukin (IL), tumor necrosis factor (TNF), and interferon (IFN) families, as well as genes associated with proliferation of lymphocytes and macrophages (MPs). Previous *in vitro* [40,41] and animal [42] studies have reported the significance of MPs at the bone-implant interface and identified favorable MP activity in relation to modified rough surfaces as demonstrated by *in vitro* gene expression that was associated with increased *in vivo* bone formation. Also, the nuclear factor-kB (NF-kB) inflammatory pathway was upregulated at day 4 [34], while macrophage activity and chemokines of the CCL and CXL families in the peri-implant tissues continued to remain prominent at day 7.

However, this inflammatory response was generally downregulated at later time points (day 7 or 14). For example, in one study, genes associated with pro-inflammatory cytokines (IL-1B, IL-1A, IL-1R2) and chemokines (CCL22, CCL18) were downregulated and upregulated, respectively, on day 7, at both implant surface technologies examined (Osseospeed and TiOBlast) [37]. Moreover, the anti-inflammatory response seemed to be modulated by surface properties. In one study, genes related to anti-inflammatory cytokines such as IL-9, IL-22, toll-like receptor inhibitor protein (TOLLIP), and several key chemokines (CCL18, CXCL10, CXCL18) were significantly upregulated on Osseospeed surfaces but not TiOBlast, at day 3 [36]. In another study, genes associated with inflammatory cell proliferation were significantly downregulated earlier on SLActive surfaces compared to the SLA, i.e., at day 7 instead of day 14 [35]. Therefore, the initial inflammatory response seems to be important for the recruitment of cells that govern subsequent healing processes and is regulated by a natural biological immune response which may be further modified by implant surface properties.

### Osteogenic differentiation

Cells along the osteogenic differentiation pathway may be artificially categorized as (1) undifferentiated MSCs, (2) osteo-chondro-progenitor cells, (3) pre-osteoblasts, and (4) osteoblasts; although in reality, a developmental continuum without distinct boundaries may exist [43]. While pre-differentiated osteoblasts in the marrow compartment only play a minor role in bone wound healing, a more prominent role is that of undifferentiated MSCs which are recruited to the regeneration site where they differentiate into osteoblasts [16]. The recruitment and differentiation of MSCs is regulated by CKs and GFs [17,19]. The GFs most commonly implicated in bone wound healing are BMPs, members of the TGF-β family, PDGF, and IGF-1 [19,20]. Moreover, the bone debris created during implant surgery, the peri-implant blood clot (i.e., platelets) and the differentiating MSCs themselves further contribute to release of GFs at the site [44,45].

All studies reported some evidence of osteogenic differentiation at an early time point (day 3 or 4) via expression of genes associated with key growth factors (bone morphogenetic proteins (BMP4, BMP6, BMP2-kinase), growth and differentiation factor-10 (GDF10), transforming growth factors (TGF-α, TGF-β), platelet-derived growth factor (PDGF), and insulin-like growth factor-1 (IGF1)), transcription factors (Runx2, Osx, Dlx3, Dlx5, Msx1, HOX genes, Sp1, Sp3), and/or osteogenic signaling pathways (TGF-β/BMP signaling, Wnt-receptors, Ras-protein/mitogen-activated protein kinase (Ras/MAPK) signal transduction). In all studies, these genes were further upregulated at day 7. Upregulation of osteogenic factors seemed regulated by implant surface. The key transcription factor osterix (Osx) was upregulated on the Osseospeed surface, but not TiOBlast at day 7 [36], while tissues adjacent to SLActive surfaces demonstrated comparatively greater BMP and Ras/MAPK expression compared to SLA surfaces at day 7 [35]. Previous *in vivo* animal studies have reported correlations between upregulated osteogenic gene expression in peri-implant tissues and enhanced histological and biomechanical measures of osseointegration during early (1- to 4-week) healing times [27,46]; nevertheless, it is unclear whether upregulation and/or overexpression of genes at a specific time point directly correlates to increased protein production *in vivo*.

The key signaling pathways, via which GFs guide osteogenic cell differentiation, are the TGF-β/BMP- and Wnt-mediated pathways [19,47]. While the BMP pathway ensures differentiation of MSCs into osteo-chondro-progenitors (OCPs), the Wnt pathway is essential for subsequent osteoblastic commitment, i.e., Wnt acts ‘downstream’ of BMP to ensure that OCPs differentiate into osteoblasts and not chondroblasts [47]. Genes associated with both TGF-β/BMP and Wnt pathway (Wnt receptors) were upregulated at day 7 [34,35] and day 14 [34] on SLA and SLActive surfaces, suggesting the occurrence of osteogenic differentiation at these time points.

GF-regulated signaling pathways exert their effects on differentiating cells via activation of TFs. The TFs Runx2 and Osx are considered as ‘master switches’ and absolute requirements for osteoblast differentiation [21] - while Runx2 is essential for MSC differentiation, Osx acting ‘downstream’ of Runx2 controls osteoblastic fate determination [48,49]. An upregulation of these genes was observed in relation to the TiOBlast, Osseospeed, and SLActive surfaces in the present review. However, at day 7, expression of Osx was significantly greater on Osseospeed than TiOBlast surfaces. This finding is consistent with previous animal [50,51] and human studies [52] where superior *in vivo* osseointegration (i.e., larger amount of bone-to-implant contact occurring earlier) of Osseospeed versus TiOBlast implants was reported. Thus, it appears that implant surface topography and/or chemistry influence peri-implant bone healing in humans both at the signaling pathway and transcription factor level.

### ECM production

Deposition of new bone on the implant surface involves the secretion of a complex ECM (scaffold) of proteins by osteoblasts, which subsequently undergoes mineralization [9]. Expression of ECM proteins is a reliable indicator of early osteogenic activity [19] and was identified in all four studies at days 7 and 14. All studies reported some evidence of ECM production and/or organization at days 7 and 14. Upregulated genes associated with ECM deposition included various collagens (Col 1 to 11), non-collagen proteins (osteopontin (OPN), osteonectin (ON), osteocalcin (OCN), bone sialoprotein (IBSP), periostin (POSTN), and ECM protein-1), alkaline phosphatase (ALP), and bone-specific adhesion proteins (integrins (ITGB4, ITGB5), laminins (LAMA2, LAMA3), and cadherins (CDH11)). Osteocalcin, the most bone-specific ECM protein and a late marker of osteogenic differentiation [19], was significantly upregulated on Osseospeed (versus TiOBlast) surfaces at day 7 [36]. Osteopontin, an ECM protein essential for mineralization [53], was significantly upregulated on SLActive comparing to SLA surfaces at day 7 [35]. The possibility that implant surface features enhance osteogenic differentiation of MSCs via upregulation of specific genes (e.g., SLActive versus SLA in regard with BMP and Wnt signaling) has been demonstrated *in vitro* [54].

Furthermore, genes associated with collagen fibril formation/organization (heat-shock protein-47 (HSP-47), pro-collagen C-endopeptidase enhancer (PCOLCE), small leucine-rich proteoglycans (SLRP)) and post-translational modification (pro-collagen lysyl-hydroxylases (PLOD1, PLOD2, PLOD3) and lysyl-oxidase (LOX)) were upregulated on Osseospeed and TiOBlast surfaces [37]. Collagen comprises approximately 90% of the ECM and collagen fibrillogenesis and organization directly determine the biomechanical properties of bone [55,56]. Genes associated with collagen fibril formation, maturation, and post-translational modification expressed by osteoblasts [57,58] were upregulated on TiOBlast and Osseospeed implants, representing early ECM organization at the bone-implant interface. These modifications determine the pattern of collagen cross-linking which in turn influences tissue organization, mineralization, and ultimately mechanical bone strength [56], and in the case of osseointegration, the integrity of the bone-implant interface [37].

### Osteoclastic activity and remodeling

While GFs regulate osteogenesis, pro-inflammatory CKs (e.g., IL-1, IL-6, TNF-α) simultaneously regulate the antagonist process of bone resorption via osteoclasts [23]. Moreover, osteoblasts themselves stimulate osteoclastogenesis via macrophage colony stimulating factor (M-CSF) and receptor activator of NF-kB ligand (RANKL) genes but also closely regulate this process via osteoprotegerin (OPG), an inhibitor of RANKL [59].

Two studies reported expression of genes associated with osteoclastic activity and ECM degradation (cathepsin-K (CTSK), tartarate-resistant acid phosphatase (ACP5), and/or matrix metalloproteinases (MMPs)), on Osseospeed and TiOBlast surfaces at day 7 [37], and SLActive surfaces at day 14. However, upregulation of MMP inhibitors (TIMP-2, -3) was also reported on TiOBlast and Osseospeed surfaces suggesting a control of the resorption process. Although no studies reported differential RANKL/OPG expression, a previous *in vitro* study [60] reported significant downregulation of osteoclastogenic genes on SLActive surfaces. Collectively, these data reaffirm the dynamic nature of bone formation and resorption at the implant-bone interface, even in early healing stages, and suggest the possibility for implant surface technology modulation of bone remodeling.

### Angiogenesis

Angiogenesis is closely related to osteogenesis and occurs simultaneously during bone regeneration [11]. Physiological oxygen tensions in bone are about 12.5% O_2_ but fall to 1% O_2_ in regeneration sites due to disruption of the local vasculature as a result of injury and/or surgery [61,62]. A key event that stimulates angiogenesis (and osteogenesis) at regeneration sites is hypoxia, via the hypoxia inducible (transcription) factor-1 (HIF-1) that regulates expression of angiogenic genes [63]. The key cells involved in angiogenesis are macrophages, which in response to hypoxia and inflammation release chemotactic and angiogenic growth factors (e.g., VEGF) [40,64], and endothelial progenitor cells (EPCs) which differentiate into endothelial cell lining blood vessels [65]. VEGF is the single most important regulator of EPC differentiation and vessel formation [66]. Moreover, a role for VEGF in osteogenic differentiation has also been suggested mainly via interaction with the BMP signaling pathway [67].

In the present review, a significant simultaneous upregulation of several angiogenesis-related genes was identified at day 7 in all included studies. Pro-angiogenic factors (ANXA2, EPAS-1) were upregulated at TiOBlast and Osseospeed surfaces at day 7 [37]. Genes associated with VEGF and P13K-AKT signaling pathways were upregulated at SLActive (but not SLA) surfaces on day 7 and continued to be upregulated on day 14 [35]. The P13K-AKT pathway is reported to be important for endothelial cell survival, migration, and vessel formation, in addition to aiding VEGF-mediated angiogenesis [68]. Previous *in vitro* studies have reported the pro-angiogenic effects of SLActive surfaces by promoting VEGF expression in EPCs and osteoblasts [65,69], while enhanced histological osseointegration of SLActive implants has been directly correlated with increased angiogenesis in a dog model [70,71]. Thus, implant surface technology appears to have the possibility to also influence angiogenesis at early stages of wound healing.

### Neurogenesis

Bone innervation includes both myelinated and unmyelinated nerve fibers located in the periosteum, bone cortex, Haversian systems, Volkmann’s canals, and the marrow spaces [72]. An interesting finding in the present review was the significant upregulation of genes associated with neurogenesis, more than any other biological process, on SLActive and SLA surfaces at all time points [34,35]. Specific processes represented were axon formation, growth and differentiation, and the neural signaling pathway. This is consistent with previous *in vivo* reports of murine fracture healing [73] and calvarial defect regeneration in relation to SLA surfaces [74,75]. Key neurotrophic factors (brain-derived neurotrophic factor (BDNF) and neurotrophin 3 (NTF3)), essential for neuronal survival and differentiation during development [76], were significantly upregulated on SLActive versus SLA surfaces at day 7 suggesting an effect of surface modulation. The P13K-AKT pathway, upregulated on SLActive surfaces (in relation to angiogenesis), has also been implicated in neuronal survival and subsequent neural development [77,78] and could have contributed to upregulation of neurogenic genes at these surfaces. Indeed, previous histologic reports have described changes in bone innervation after implant placement (and loading) and the presence of nerve fibers within the peri-implant bone, in animals and humans [79-81].

It can be hypothesized that peri-implant neurogenesis is one of the underlying mechanisms governing the phenomenon of osseoperception, defined as the tactile sensibility of osseointegrated implants to occlusal forces induced via activation of nerve endings and/or receptors in the peri-implant environment [82,83]. Moreover, recent evidence suggests that implant surface properties may influence the degree of osseoperception in humans [84], which can be correlated with the genomic evidence for implant surface modulation of neurogenesis during osseointegration.

Finally, the present review findings are consistent with a recent gene expression study of healing extraction sockets in humans [85]. This study reported an initial upregulation of pro-inflammatory cytokines (IL-1, IL-6) at day 1, but by day 7, genes suggestive of immune response (IL-10), osteogenesis (TGF, BMP4, BMP7, OCN and ALP), and angiogenesis (VEGF) were upregulated, continuing until day 14, suggesting that the basic biological processes governing alveolar wound healing and osseointegration are the same.

## Conclusions

Based on limited evidence of gene expression data from four studies involving 43 patients, the following remarks can be made:

1. Early peri-implant healing (2 weeks) involves a sequence of biological events which are similar to those observed in other bone wound healing scenarios (fractures, extraction-sockets).
2. Osseointegration depends on osteogenesis at the implant interface, but other simultaneously occurring processes such as inflammation, bone resorption, angiogenesis and neurogenesis also play an important role, as evidenced by consistent and concomitant gene expression.
3. Several genes associated with key regulators of biological processes, such as cells, cytokines, growth factors, transcription factors, signaling pathways, and secretory products, were shown to be differentially regulated during peri-implant healing in a manner that was largely consistent - in terms of nature and timing - with previous *in vitro* and preclinical *in vivo* histological studies of osseointegration.
4. Implant surface technology can influence osseointegration, at every step of the early wound healing process, i.e., anti-inflammatory response, progenitor cell recruitment, osteoinduction, growth factor/transcription factor expression, signaling pathway regulation, and extracellular matrix production. However, the relevance of those observations is questionable; no distinct differences have been demonstrated in terms of histological outcomes at later time points or short- and long-term clinical performance among the various implant surface technologies discussed herein.
